# Electromyographic comparison of the barbell deadlift using constant versus variable resistance in healthy, trained men

**DOI:** 10.1371/journal.pone.0211021

**Published:** 2019-01-22

**Authors:** Vidar Andersen, Marius S. Fimland, Dag-Andrè Mo, Vegard M. Iversen, Tommy M. Larsen, Fredrik Solheim, Atle H. Saeterbakken

**Affiliations:** 1 Faculty of Education, Arts and Sport, Western Norway University of Applied Sciences, Sogndal, Norway; 2 Department of Neuromedicine and Movement Science, Faculty of Medicine and Health Sciences, Norwegian University of Science and Technology, Trondheim, Norway; 3 Unicare Helsefort Rehabilitation Centre, Rissa, Norway; 4 Department of Public Health and Nursing, Faculty of Medicine and Health Sciences, Norwegian University of Science and Technology, Trondheim, Norway; Hochschule Trier, GERMANY

## Abstract

Variable, external resistance is proposed to increasingly augment the muscular stress throughout a dynamic movement. However, it is uncertain how different levels of variable resistance affect the activation in the deadlift. The aim of the study was to compare the electromyographic activity of the gluteus maximus, biceps femoris, semitendinosus, vastus lateralis and erector spinae muscles during the barbell deadlift with free weights (FW) alone, with two (FW-2EB), and four elastic bands (FW-4EB) to deload some of the constant external resistance. Fifteen resistance-trained men participated in a cross-over design where resistance loadings were matched using two-repetition maximum loadings in the three different conditions. For the whole movement, both repetitions were analyzed. For the phase-specific analysis, the last repetition was divided into six parts, i.e. the lower, middle and upper phase in both the ascending and descending phase of the movement. The mean deloading contributions from FW-2EB and FW-4EB were 21% and 41%, respectively. In FW-4EB, the erector spinae was activated more in the whole movement (8%, ES = 0.31, *p* = 0.002) compared to FW-2EB. There was also a tendency towards higher activation in FW-4EB versus FW for the whole movement (5%, ES = 0.18, *p* = 0.072). There were no significant differences between the conditions in any of the other phases or muscles (*p* = 0.106–0.926). In summary, a high contribution from variable, external resistance seems to activate the back extensors more than a low contribution.

## Introduction

Resistance training exercises using free weights, such as a barbell, provides constant external mass throughout the movement. Consequently, whether a lift can be successfully completed or not depends on the lifter`s maximal strength in the so called”sticking region” where there is a disproportionately large increase in the difficulty to complete the lift [1–3]. A muscles`ability to produce force changes throughout a movement mainly due to the shifting overlap of actin and myosin filaments. Free weights have been combined with chains or elastic bands in attempts to match the external torque requirements to the torque-generating capacity of the muscles throughout the range of motion [4, 5]. In theory, this should increase muscle activation in the range of motion beyond the sticking region [6].

In the barbell squat exercise, several studies have compared the acute effects of constant external versus variable external resistance on neuromuscular activation [4, 5, 7, 8]. Studies utilizing a relatively small contribution from variable external resistance (10% or less) did not find significant differences in muscle activation [5, 7]. However, a higher contribution from variable resistance (e.g. 20–40%) seems advantageous for muscle activation, in comparison with constant resistance only, or low levels of variable external resistance [4, 8]. Further, in line with the theory of increased muscle activation beyond the sticking region, the two studies that divided the movement into different phases showed variable external resistance to be advantageous in the upper parts of the movement [4, 8].

Comparatively less research is done on the barbell deadlift which is another of the three lifts in powerlifting, and also a popular exercise among bodybuilders, athletes and recreationally active individuals seeking to increase muscle hypertrophy and general strength [9]. Despite the similarities between the squat and the barbell deadlift—both being compound knee- and hip-extensor exercises—the biomechanics between the two exercises are quite different, with the barbell deadlift requiring comparatively more effort from the hip extensors [10]. In a study by Nijem et al. [11], 13 resistance-trained men performed deadlift with either a barbell or barbell + chains to add variable resistance. The authors reported no differences in muscular activation for the erector spinae or the vastus lateralis between the two conditions, but a 7% higher activation of the gluteus maximus for constant external resistance. No studies have yet investigated muscle activation in different phases of the deadlift. In addition, it was shown that different levels of variable resistance alters the kinetics of the deadlift [12], which could impact the muscle activation. Galpin et al. [12] showed that variable resistance increased power and velocity, but decreased maximal force in explosive deadlifts with moderate and high intensities (60 and 85% of 1-RM).

**Fig 1:**
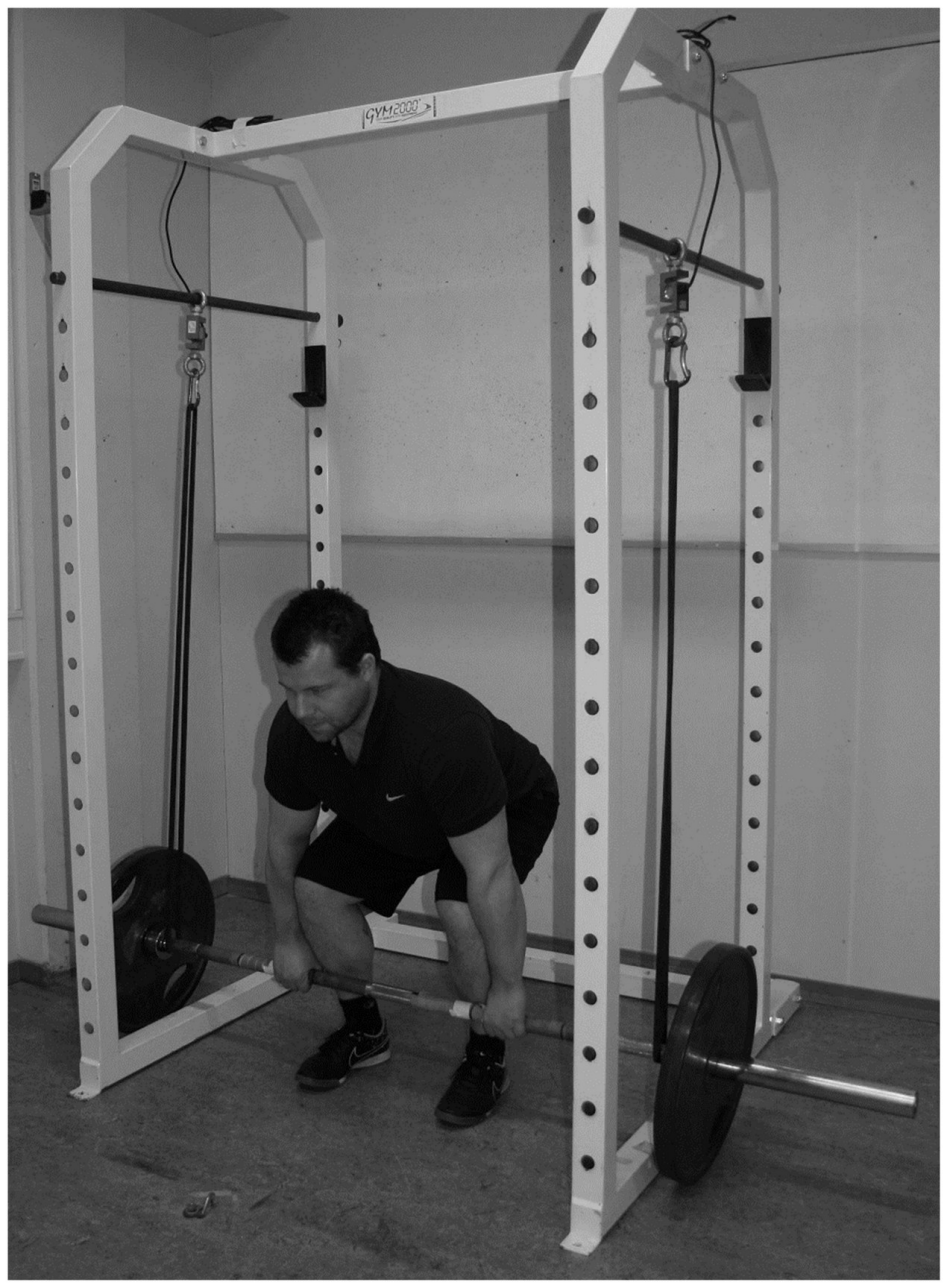
Lower position in the traditional deadlift. Picture showing execution with free weights—two elastic bands.

Previous studies have added variable resistance to the barbell load in squats and deadlift [7, 8, 11]. Another popular methods of variable resistance during deadlift exercises is to attach elastic bands above the barbell (see Fig 1), often called reverse band deadlift. This allows for more free weights to be added to the bar as the elastic bands deload the free weight loading most in the beginning of the lift, and gradually less throughout the movement. Elastic bands have shown to create a curvilinear tension-deformation relationship when being stretched [13]. That is, the contribution from the variable resistance will not increase linearly with the lengthening of the elastic bands. Therefore the deload set-up could thus create a potential for higher resistance in the upper parts of the movement since the constant resistance contributes increasingly to the total load as the bands are shortening. However, we are not aware of studies investigating effects of different levels of muscle activity of reverse band deadlift.

The aim of this study was to compare muscle activation during the barbell deadlift using either free weights (FW), FW and two elastic bands (FW-2EB) or FW and four elastic bands (FW-4EB) to deload the free weights, using matched relative resistance (2 Repetition Maximum, 2-RM). Based on results from investigations on the squat [8], we hypothesized higher activations in the upper phase of the lift with variable external resistance versus FW only, particularly for FW-4EB.

## Methods

### Experimental approach

A randomized, cross-over design was used to examine the electromyography activity (EMG) in gluteus maximus, semitendinosus, biceps femoris, erector spinae and vastus lateralis muscles in conventional barbell deadlifts with FW, FW-2EB (one band at each side of the bar) and FW-4EB (Fig 1). The same relative intensity (2-RM) was used in each condition. The elastic bands were used to deload the weights, providing less resistance from the barbell in the lower phase of the movement and increasingly more as the barbell was lifted through the middle and the upper phase of the movement. To ensure reliable EMG data, all testing involving EMG was executed in one session in a randomized and counter-balanced order.

### Subjects

Fifteen healthy resistance-trained men (age 23.3 ± 2.2 years, body mass 82.8 ± 11.1 kg, stature 182 ± 6 cm, 3.9 ± 1.9 years of resistance training experience, 2-RM deadlift 154 ± 28 kg) volunteered for the study. All participants trained the conventional barbell deadlift on a weekly basis in the last year. The participants had some experience in training with variable external resistance, but none regularly used elastic bands in their deadlift training. Moreover, none were competing in power- or weightlifting. Exclusion criteria were musculoskeletal pain, unfamiliarity with the deadlift exercise, injury or illness (e.g. hamstring strain) that might reduce maximal effort, having less than six months of resistance training experience or being under 18 years old. All participants were instructed to refrain from alcohol and resistance training 72 hours prior to the two testing sessions. All participants were informed verbally and in writing of the procedures and possible risks of the tests and provided written consent before they were included in the study. The individual in this manuscript has given written informed consent (as outlined in PLOS consent form) to publish these case details. The study conformed with the latest revision of the Declaration of Helsinki and the ethical guidelines at the Sogn og Fjordane University College and was approved by the Norwegian Centre for Research Data before the start of the study.

### Procedures

To become familiar with the exercises and establish the 2-RM for the three conditions, all participants had to attend two familiarization/strength testing sessions before the experimental session, separated by 2–7 days. The warm-up was identical in all sessions: 1) five minutes on a bicycle or a treadmill, 2) 12 repetitions at 40% of 2-RM, 3) 8 repetitions at 60% of 2-RM, and 4) 5 repetitions at 85% of 2-RM using the barbell deadlift (FW). In the first familiarization session, the 2-RM load used to calculate the warm-up loads were self-reported by the participants. In the second familiarization and the experimental tests, the 2-RM load from the respective previous test was used. The intraclass correlation coefficient and coefficient of variation of the 2-RM loads between the second familiarization and the experimental test varied from 0.98 to 0.99 and from 1.7 to 2.0%, respectively.

**Fig 2:**
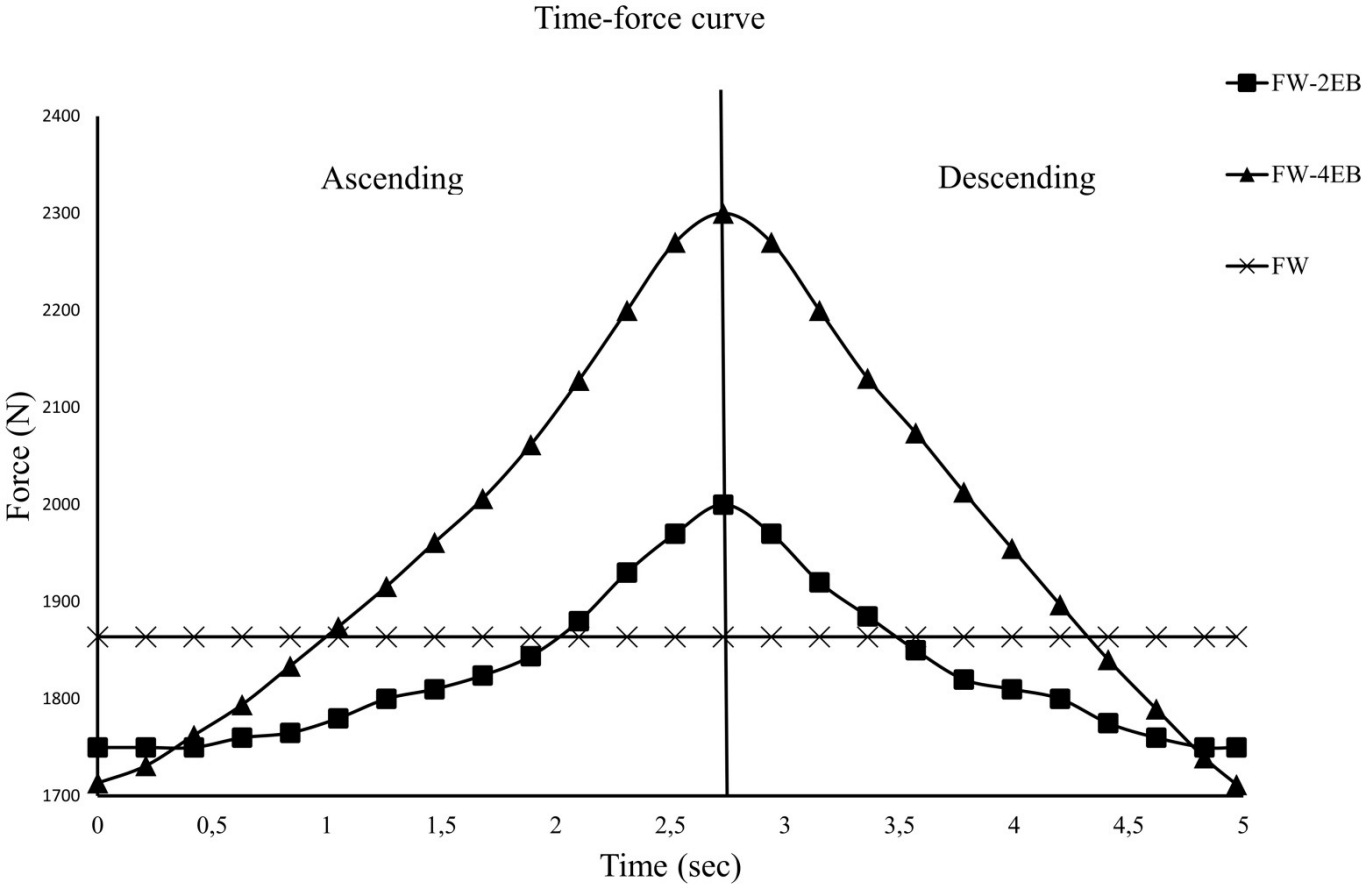
Example of the total external load during one repetition in the three conditions. The data are collected from one person.

The lifting was performed in a power rack (Gym 2000, Modum, Norway) with a barbell (20 kg), weight plates and elastic bands (dimension; 1 cm (width) x 0.5 cm (height), Ropes 302, Bungee, Norway) (Fig 1). The participants selected their preferred grip width and feet position (width and orientation) in the first session. These were recorded and replicated in all subsequent lifts. Lifting straps and chalk were allowed to avoid grip strength being a limiting factor. The participants chose to use either no shoes or lifting shoes (same in all conditions for each individual). The participants performed the repetitions in a controlled, but self-selected tempo. The lift started with the weights resting on the floor. The participants were instructed to lift the barbell while maintaining a straight back and to extend their knees and hip in one movement (to avoid a straight-leg deadlift-technique). The ascending phase was complete when the hip and knees were fully extended. The participants then reversed the movement, lowering the weight to the floor in a controlled manner (no bouncing), then the second repetition was performed. No pauses were allowed in the upper or lower position. During the variable, external resistance conditions, two or four elastic bands were attached between the barbell and two force cells (Ergotest Technology AS, Langesund Norway) allowing the unloading from the elastic bands to be monitored through the whole movement (Fig 1). To assess the total load in each condition, the free weights (bar + plates) were measured with the bar laying still (mass x gravity). For the FW-2EB and FW-4EB conditions, the force contribution from the elastic bands were subtracted. The height of the force cells were adjusted for each person so the contribution of the elastic bands should be approximately 25% (2EB) and 50% (4EB) of the 2-RM load in the lower position. An example of the total load throughout the movement in the three conditions is shown in Fig 2.

After the warm-up in the experimental test, the 2-RM loads lifted in the second familiarization test were used as the starting load. The load was then increased or decreased by 2.5 kg or 5 kg until 2-RM was achieved (1–3 attempts). Before the test for each condition, the participants were given one familiarization set consisting of four to six repetitions with a submaximal load (50% of the 2-RM loads), to adjust the technique to the new test. The 2-RM testing was terminated when the participants failed to complete a lift, could not complete the lift with proper technique or both the participant and test leader agreed that the participant would not be able to lift 2.5 kg more. The highest weight lifted in each condition was defined as its`2-RM.

### Electromyography

Before the experimental tests, the skin was prepared (shaved, washed with alcohol, and abraded) before placement of gel coated self-adhesive electrodes (Dri-Stick Silver circular sEMG Electrodes AE- 131; NeuroDyne Medical, Cambridge, MA, USA) (11,21). The electrodes (11-mm contact diameter) were placed along the presumed direction of the underlying muscle fibers with a center-to-center distance of 2 cm according to the recommendations by SENIAM [14]. The electrodes were placed on the dominant leg (defined as the leg used to kick a ball). The electrode on the gluteus maximus was placed at half the distance between the sacral vertebrae and the greater trochanter. The electrode on the semitendinosus was placed at half the distance between the ischial tuberosity and the medial epicondyle of the tibia. The electrode on the biceps femoris was placed at half of the distance between the ischial tuberosity and the lateral epicondyle of the tibia. The electrode on the erector spinae was located at L1, three centimeters lateral to the spinous process. Finally, the electrode on vastus lateralis was located two thirds down the line between spina iliaca anterior superior and the lateral side of the patella.

For the analysis of the whole movement, the root-mean-square (RMS) EMG obtained during both repetitions was used. To minimize noise from the surroundings, the raw EMG signal was amplified and filtered using a preamplifier located close to the sampling point. The preamplifier had a common mode rejection ratio of 100 dB, high cut frequency 600 Hz and low cut frequency 8 Hz. The EMG signals were converted to RMS using a hardware circuit network (frequency response 0–600 kHz, averaging constant 100 ms, total error ± 0.5%). Finally, the RMS converted signal was sampled at 100 Hz using a 16 bit A/D converter. Commercial software (MuscleLab V8.13, Ergotest Technology AS, Langesund, Norway) was used to analyze the stored EMG data.

For the phase-specific analysis, the last repetition was divided into the lower, middle and upper phase of the ascending and descending movement. The classification of each phase was made from the trajectory of the barbell, dividing the total distance into three identical parts. To identify the beginning and the end of the lift, as well as the different phases and the lifting time, a linear encoder was attached to the barbell (Ergotest Technology AS, Langesund Norway, sampling frequency of 100 Hz). The linear encoder was synchronized with the EMG recording system (MuscleLab 4020e, Ergotest Technology AS, Langesund, Norway).

### Statistical analyses

We used linear mixed-effects models to compare muscle activation in the three experimental conditions, i.e. 1) FW, 2) FW-2EB, 3) FW-4EB. Each muscle was analyzed separately, with its EMG activity included as the dependent variable. Contraction phases and resistance modalities as well as their interaction terms, was included as fixed effects while participant identity was included as a random effect (i.e. to allow for different starting points). The distribution of all regression residuals were examined and found to be normally distributed. Lifting time was considered as a potential confounder and all analysis were therefore performed both with and without adjustment for this variable. Statistical analyses were done using STATA/IC 13.1 for windows (StataCorp LP, USA).

Differences in lifting time and total loading were assessed with one-way repeated measures analyses of variance with Bonferroni as post hoc-test where appropriate. These analyses were performed with SPSS version 17.0 (SPSS, Inc., Chicago, IL, USA).

The significance level for main effect and interaction effect was set to *p*<0.05. Precision was assessed using 95% confidence intervals. Cohen`s *d* was used to calculate the effect size (ES). An ES of 0.2 was considered small, 0.5 medium and 0.8 large [15].

## Results

Throughout the range of motion, the mean contribution from the elastic bands to the total load was 21% for the FW-2EB and 41% for the FW-4EB. Mean contributions from elastic and constant resistance is presented in Table 1.

**Table 1:** Total external resistance and contribution from constant resistance (free weights) in the lower-, middle- and upper phase for the three experimental conditions.

	Lower phase	Middle phase	Upper phase	Constant resistance
Free weights	1512 ± 277 N	1512 ± 277 N	1512 ± 277 N	1512 ± 277 N
Free weights—two elastic bands	1429 ± 300 N	1530 ± 294 N	1634 ± 286 N	1830 ± 294 N
Contribution (deload) from elastic bands	29 ± 7%	20 ± 4%	12 ± 3%	
Difference from free weights	-6 ± 7%	1 ± 6%	8 ± 7%	22 ± 8%
Free weights—four elastic bands	1409 ± 316 N	1591 ± 312 N	1774 ± 310 N	2197 ± 312 N
Contribution (deload) from elastic bands	59 ± 14%	40 ± 8%	25 ± 5%	
Difference from free weights	-7 ± 8%	5 ± 8%	18 ± 9%	47 ± 11%

Values are newton (N) or percentage (%). Means ± standard deviations.

For the whole movement, the FW-4EB (1592 ± 312 N) provided 4% higher total loading compared to the FW-2EB (1531 ± 293 N, *p* = 0.002) and a tendency towards a 5% higher loading compared to FW (1512 ± 277 N, *p* = 0.059). There were no statistical differences between FW-2EB and FW (*p* = 0.490).

**Fig 3:**
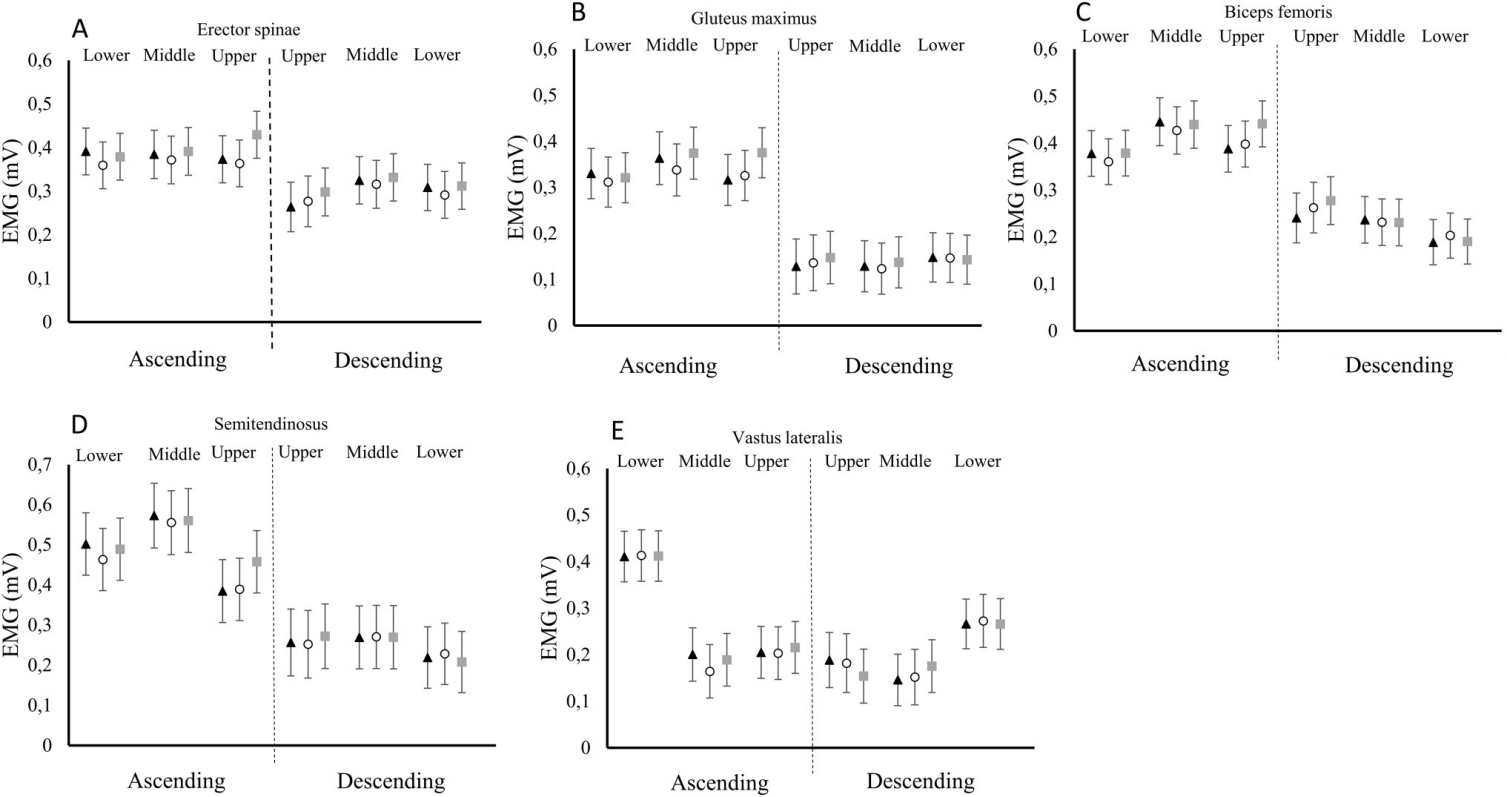
A-E: Mean EMG activity in the different phases of the movement in erector spinae (A), gluteus maximus (B), biceps femoris (C), semitendinosus (D) and vastus lateralis (E) during traditional deadlift with free weights (Δ), free weights—Two elastic bands (○) and free weights—four elastic bands (◻). Values are means with 95% CI.

No interaction effects were displayed between the exercise modalities and contraction phases for the erector spinae muscle (*p* = 0.570). However, tests of main effects demonstrated an 8% higher activation of the erector spinae for the FW-4EB compared to the FW-2EB for the whole movement (ES = 0.31, *p* = 0.002, Table 2) and a tendency towards higher activation in the FW-4EB versus FW (5%, ES = 0.18, p = 0.072). There was no difference between FW-2EB and FW (*p* = 0.193). No interactions (*p* = 0.726–0.926) nor main effects (*p* = 0.106–0.913) were displayed for any of the other muscles (Table 2 and Fig 3).

**Table 2:** Neuromuscular activation for the whole movement.

	Free Weights	Free Weights—2 elastic bands	Free Weights—4 elastic bands
Erector spinae	0.341 (0.295–0.388)	0.330 (0.283–0.376)	0.357 (0.311–0.403)*
Gluteus maximus	0.236 (0.196–0.276)	0.231 (0.190–0.271)	0.250 (0.210–0.290)
Biceps femoris	0.312 (0.275–0.350)	0.313 (0.276–0.351)	0.326 (0.289–0.363)
Semitendinosus	0.367 (0.304–0.430)	0.359 (0.296–0.422)	0.375 (0.313–0.438)
Vastus lateralis	0.239 (0.198–0.280)	0.234 (0.192–0.276)	0.238 (0.197–0.279)

* *p* ≤ 0.01 compared to two elastic bands

Electromyogram root-mean-square (mV) values are presented as means ± 95% confidence intervals.

On average, there were similar lifting times for the different conditions in the total lifting time (means ± SD): FW: 10.13 ± 1.06 sec, FW-2EB: 10.40 ± 1.78 sec, FW-4EB: 10.32 ± 1.61 sec (*p* = 0.496–0.801) and time per repetition: FW: 4.34 ± 0.77 sec, FW-2EB: 4.38 ± 0.77 sec, FW-4EB: 4.00 ± 0.61 sec (*p* = 0.128–1.000). However, as the inclusion of lifting time as a covariate resulted in more than 10% change in the regression coefficients, it was adjusted for in the final analysis. No conclusions were altered by this adjustment.

## Discussion

The main findings of the study were that the overall activation of the erector spinae was 8% higher for FW-4EB compared to the FW-2EB (ES: 0.31). Furthermore, the erector spinae tended to be more activated during the FW-4EB than the FW in the whole movement (5%, ES: 0.18). No other meaningful differences were observed.

The present study used elastic bands to deload the weights in contrast to most previous studies that used elastic bands to add load [7, 8, 12]. Both ways of introducing variable resistance achieves the same overall purpose in the deadlift: making the exercise easier in the bottom and more challenging near the end-range of the motion, compared to constant external resistance. In the start of the stretch, an elastic band will increase the tension/load linearly. However, at a specific length of the elastic band, tension will level off during stretching creating a curvilinear tension-deformation relationship [13]. In other words, when the elastic bands are attached to the floor and stretched as the bar is lifted, the contribution from the variable resistance will not increase linearly with the lengthening of the elastic bands. If the elastic bands are used to deload the barbell this will apply to the lower parts of the movement since it is here the bands are stretched the most. Using this set-up will therefore create a potential for higher resistance in the upper parts of the movement since the constant resistance contributes increasingly to the total load as the bands are shortening. Further, the deload set-up could have a possible benefit compared to the load set-up in the lower parts of the movement as well, making the constant and the variable condition more similar due to the same curvilinear tension-deformation relationship. This speculation is supported by the force data presented in Table 1. They state a clear force hierarchy in the upper parts of the movement between the conditions (FW-4EB > FW-2EB > FW). In the lower phase the ranking is reversed, but the differences are more subtle. This could explain why the present study found differences in the erector spinae activation during the whole movement, while a previous study using bands to increase load, did not [7]. Importantly, unlike us Saeterbakken et al. [7] did not find any differences between the constant and variable modality for the whole movement. This could be a result of the elastic bands contributing less to the total resistance in the middle (35%) and upper (42%) phases compared to the present study.

The increased total resistance throughout the movement could explain why the erector spinae activation was greater during FW-4EB than FW-2EB, but not why the activation during FW-4EB only tended to be greater than the FW. Although there was only was a tendency towards difference in load for the whole movement between FW-4EB and FW, the difference between FW-4EB and FW was more pronounced in the upper phase (18% higher loading for FW-4EB, ES = 0.89). That this relatively large percentage difference in loading with a corresponding high effect size did not result in a statistically significant difference in muscular activation could be due to individual differences in how participants adjusted their lifting strategy when the barbell deadlift, a habitual task, was modified by applying elastic bands. The incorporation of elastic bands have been shown to affect the kinetic and kinematics of an exercise, and thus influence the movement [6, 12, 16]. The seemingly subtle change in lifting technique may have affected participants differently. While we anticipated a dose-response activation pattern of EMG activity in the upper phase of the lift (i.e. FW-4EB > FW-2EB > FW), it could be that the deloading from elastic bands was too low in the FW-2EB to compensate for the altered motor control demands induced by elastic bands. Further, regardless of loading modality, the high loading in all conditions (2-RM) would require near maximal effort. It could be speculated that a pre-planned high level of effort in some individuals may have led to very high activation levels throughout the range of motion, regardless of differing demands throughout the movement in the different conditions. That is, that they were less susceptible to proprioceptive feedback. This notion lends support from limb blocking studies showing that the neuromuscular activation can be upheld for a period of time even after a limb has been unexpectedly mechanically blocked [17]. The participants in the present study did not use elastic band in their regular training routine. Recruiting lifters more experienced in elastic band-training [12, 18], might have provided findings more in line with our expectations of a dose-response pattern.

The finding of no significant differences in activation of the knee- or hip extensors between the conditions were expected for the whole movement, but not for the upper phase where differences in external loading were more profound. In the upper phase, the load between the different conditions varied from 8–18% with effect sizes varying from small to large (ES = 0.43–0.89). This should hypothetically increase the neuromuscular stress by improving the muscle length-force relationship in this part of the movement. Nevertheless, the EMG activation was usually non-significantly higher for the elastic band conditions in the ascending phase, in what appears to be a dose-response relationship (Fig 3A–3D). Therefore, it is possible that the differences would have reached statistical significance, if the differences in variable loading had been greater. In addition, it could be due to limited statistical power resulting in type II-errors. Although the number of participants (i.e. 15) is similar to comparable studies [11, 12], it is possible that more statistical differences would be detected had more participants been recruited.

One previous study compared muscle activation levels between constant external and variable, external resistance in the barbell deadlift [11]. Partly in line with our results, Nijem et al. [11] found no significant differences in activation of the vastus lateralis muscle for the whole movement, when they compared barbell deadlift performed with and without added chains. However, they observed no difference in the erector spinae but a 7% higher gluteus maximus activation using constant, external compared to variable external resistance. This could be explained by methodological differences as we compared conditions with the same relative intensity (2-RM), while Nijem and co-workers used a more demanding constant, than variable external loading condition. In part, it could also be due to the different properties between elastic bands and chains and/or introducing external resistance through adding vs deloading weight.

Two other studies, with modest differences in the variable, external resistance-loading component, also reported similar activations during constant external and variable external resistance in the squat [5, 7]. However, a study from our lab [8], using a greater contribution from variable, external resistance in the squat, showed higher activation, especially in the upper ascending phase, of the quadriceps and hamstring muscles in the external, variable condition. Although the deadlift and squats have different biomechanical requirements [10], the contribution from the variable, external resistance in the present study (21 and 41%) was substantially lower than in our study investigating the squat (39 and 73%) [8], which could explain the different findings.

Some limitations must be acknowledged. Although all participants took part in two familiarization sessions, none used elastic bands on a regular basis in their deadlift workout routine. It is possible that recruiting more experienced lifters would have provided the dose-response pattern we expected for muscle activation in the upper phase (FW-4EB > FW-2EB > FW). There will always be a potential for crosstalk from the neighboring muscles when using surface EMG [19]. There are also some additional methodological challenges when assessing EMG during dynamic (instead of isometric) contractions, such as electrode shift relative to the origin of muscle action potentials and changes in conductivity of the tissues separating muscle fibers and electrodes [20]. These factors could create random noise; reducing the power to detect true differences between the conditions. Had more participants been recruited, the statistical power would have been increased. A strength is that all EMG data was collected in one session without having to replace the electrodes, as previously recommended [21]. Another limitation is that the force created from the acceleration of the bar was not included when calculating the total load. Previous studies have shown that adding elastic bands alter kinetic variables such as velocity, power and force [12, 16]. However, participants were instructed to lift in a controlled manner, and the lifting times were similar between conditions. Finally, very heavy loading (2-RM) was used in all tests and it is possible that the relative contribution from the muscles involved would have differed with submaximal loadings.

## Practical applications

Applying elastic bands to a barbell introduces variable, external resistance throughout the range of motion, which could help the lifter to overcome or displace the sticking point, the bottleneck of the concentric movement in constant external resistance training. Hence, variable, external resistance could increase the neuromuscular stress in the phases near the end-range of the movement. In this study, the contribution from variable, external resistance was 9% and 17% in the upper phase of the movement. The condition with the highest contribution from variable, external resistance induced higher activation of the erector spinae, whereas there were no other significant differences. These results were obtained in resistance-trained individuals, but who did not regularly train barbell deadlift accommodated with elastic bands. Thus, our findings may or may not apply to individuals with more experience with the exercise.

Whether higher activation of the lower back muscles, as we observed with a high contribution from elastic resistance, is beneficial, would depend on the goal of a particular training session. In addition, previous studies have shown that a high contribution from the elastic bands increases power and velocity compared to free weights, especially when lifting at high intensities [12, 16], although force production is reduced [12]. Therefore, athletes and strength coaches should consider which variable to emphasize in each session before choosing to use constant resistance only, or introduce variable resistance.

## Conclusion

In conclusion, the barbell deadlift with FW-4EB produced the greatest erector spinae activation. A high deloading contribution from elastic bands in the barbell deadlift, allowing for higher total external loading, particularly in the upper phases, induced greater neuromuscular activation of the erector spinae than a small deloading contribution. For the hip and knee extensors, there were similar activations across the different conditions. If coaches and practitioners want to emphasize lower back activation, we recommend a relatively high contribution (e.g. 40%) from elastic bands to the total resistance.

## Supporting information

S1 File EMG. EMG data.
(XLSX)

S2 File Load. Load data.
(XLSX)
